# Promising Biomarkers for Spinal Cord Injury: A Brief Review

**DOI:** 10.19080/ctbeb.2024.22.556095

**Published:** 2023-04-25

**Authors:** Anita Singh

**Affiliations:** Associate Professor, Bioengineering, Temple University, PA, USA

**Keywords:** Spinal cord injury, Biomarkers, Neuronal injury, Structural biomarkers, Inflammatory biomarkers, Animal studies, Human studies

## Abstract

Mechanical trauma to the spinal cord leads to neural and molecular pathophysiological responses. Biomarkers are emerging as a promising unbiased tool that can assess the pathophysiological processes following spinal cord injury (SCI). In this concise review, we provide details of role and expression of structural, including Neurofilament Proteins (NF), Glial Fibrillary Acidic Protein (GFAP), Cleaved Tau (C-Tau), S100-β, Neuron Specific Enolase (NSE), Myelin Basic Protein (MBP), and inflammatory, including Transforming Growth Factor Beta (TGF-β), Tumor Necrosis Factor (TNF), Interleukins (ILs), Soluble CD95 Ligand (sCD95L) biomarkers in reported human and animal SCI studies.

## Introduction

Spinal cord injury leads to paralysis in over 10,000 individuals annually in the US [1,2]. Mechanical trauma to the spinal cord leads to neural and molecular pathophysiological responses including the disruption of axons, the surrounding glial cells and blood vessels [3–5]. Current gold standard of injury severity includes functional assessment. However, an early diagnosis of SCI warrants investigation of biological markers that can inform, both the injury severity and its outcome. Biomarkers are emerging as a promising unbiased tool that can assess as well as predict the pathophysiological processes following SCI. Widely accepted definition of biomarker is: “A defined characteristic that is measured as an indicator of normal biological processes, pathogenic processes or responses to an exposure or intervention” [6]. Reported structural biomarkers for SCI include Neurofilament Proteins (NF), Glial Fibrillary Acidic Protein (GFAP), Cleaved Tau (C-Tau), S100-β, Neuron Specific Enolase (NSE), Myelin Basic Protein (MBP). Additionally, SCI inflammatory biomarkers include Transforming Growth Factor Beta (TGF-β), Tumor Necrosis Factor (TNF), Insulin-Like Growth Factor (IGF), Interleukins (ILs) and Soluble CD95 Ligand (sCD95L). In this review we provide an overview of these biomarkers.

## Structural Biomarkers

### Neurofilament Proteins (NFs)

Neurofilaments (NFs), a cytoskeletal protein, are found abundantly in the cytoplasm of central nervous system (CNS) axonal fibers and regulates axonal transport and signaling [7,8]. Extracellular accumulations of NFs are widely reported in multiple neurological pathologies including SCI [9]. During traumatic SCI, NFs are released from the cytoplasm of the damaged neurons serving as an indicator of extent of neuronal loss in SCI. A clinical study in subjects with varying degree of motor loss reported elevated levels of NFs in the serum to be associated with more severe SCI and poorer neurological prognosis [10].

### Glial Fibrillary Acidic Protein (GFAP)

GFAP is an intermediate filament protein that is responsible for development of the cytoskeleton of astroglial cells and is upregulated during injury by these cells [11]. Recent studies have confirmed GFAP as an established biomarker of central nervous system [7,12]. In a clinical study, SCI patients reported GFAP as a strong predictor of AIS graded injury severities at 24 h as well prognosis as predicted 6-month postinjury segmental motor improvements [1].

### Cleaved Tau (C-Tau)

Tau is a Microtubule Associated Protein (MAP) that plays a role in axonal transport and maintains the stabilization of axons [7]. Post SCI, high concentration of C-Tau has been reported in both the sera and CSF resulting from disrupted axons [12].

### S100-β

S100-β is a calcium binding protein found in glial cells that helps maintain homeostatic activities such as regulation of calcium fluxes, cell proliferation and differentiation, enzymatic/metabolic activity, and stabilization of MAPs [7,12]. Elevated levels of S100-β has been reported in serum levels 6 h after injury of contused SCI animals, however 24 h post-injury, these levels returned to those reported in sham [13]. Limited studies investigating S100-β protein warrant future clinical and animal studies that can offer temporal and spatial expression of this biomarker post injury.

### Neuron Specific Enolase (NSE)

NSE belongs to the glycolytic enzyme enolase family and is found primarily in neuronal cytoplasm. Axonal injury studies have reported high concentrations of NSE to reestablish cellular homeostasis [12]. Loy et al. [14] reported high levels of both S100-β and NSE in the serum at both 6 h however, NSE only at 24 h when compared to sham animals [13]. In another clinical study conducted at multiple timepoints, the levels of NSE proteis was significantly correlated with the severity of the injury [14].

### Myelin Basic Protein (MBP)

Myelin Basic Protein (MBP) is responsible for the formation and maintenance of the structure of the compact myelin sheath [12]. Swine SCI model reported elevated levels of MBP in the tissue, CSF and serum levels over a 3-h period post injury [15].

## Inflammatory Biomarkers

### Transforming Growth Factor Beta (TGF-β)

TGF-β is a polypeptide that is known to regulate stem cell differentiation, recovery processes, inflammation, and immune responses, and embryogenesis. Clinical studies have reported higher levels of TGF-β post injury while serving as a subacute inflammatory marker [16].

### Tumor Necrosis Factor (TNF)

TNF-Alpha is reported as a strong indicator of SCI pathology. A known proinflammatory cytokine that is expressed within a few hours after injury, TNF-Alpha is a widely reported inflammatory biomarker [17]. A significant decrease in TNF-Alpha levels have been reported in SCI patients that reported an improvement in Impairment scale (AIS) grade [18].

### Interleukins (ILs)

Interleukins (ILs) are cytokines that help regulate and stimulate immune function and growth. They are produced by leukocytes and are reported to increase inflammation and glial scar tissue formation post-SCI [19]. Kwon et al. reported CSF levels of IL-6 and IL-8 in SCI patients and predicted patient improvements of an AIS grade over 6 months after injury. These studies support ILs levels as a strong inflammatory biomarker post-SCI [1].

### Soluble CD95 Ligand (sCD95L)

In the subacute stages of SCI, sCD95L is involved in the induction of the extrinsic apoptotic cascade. Both animals and human studies have reported the upregulation of CD95 receptor production on the oligodendrocytes and spinal cord neurons. In a clinical study, Biglari et al. confirmed a drop in the serum levels of sCD95L at 4, 9, 12, and 24 h postinjury, while the levels increased at 8 and 12 weeks [20].

## Conclusion

Current clinical and animal studies support SCI biomarkers as a promising quantitative measure of the biochemical, physiological or behavioral changes occurring in response to injury. Future studies are needed to match a biomarker with an injury event to further their role as diagnostic and prognostic tools. In summary, biomarkers can offer a measurable tool to not only understand of the acute pathophysiology of human SCI but also guide the scientific development of novel therapies that can help improve SCI outcomes.

## Abbreviations:

SCI: Spinal Cord Injury
NFs: Neurofilaments
GFAP: Glial Fibrillary Acidic
C-Tau: Cleaved Tau
NSE: Neuron Specific Enolase
MBP: Myelin Basic Protein
TGF-B: Transforming Growth Factor Beta
TNF: Tumor Necrosis Factor
ILs: Interleukins
sCD95L: Soluble CD95 Ligand
CNS: Central Nervous System
AIS: Impairment Scale
